# Cigarette smoke-induced exosomal miR-221-3p facilitates M1 macrophage polarization via the STAT3 pathway in chronic obstructive pulmonary disease

**DOI:** 10.18632/aging.206095

**Published:** 2024-08-29

**Authors:** Hui Jia, Wei He, Bo Wu, Zhaoshuang Zhong, Yuele Chang, Yang Liu, Min Wang, Shuyue Xia

**Affiliations:** 1 Graduate School of Dalian Medical University, Dalian, China; 2 Department of Respiratory and Critical Care Medicine, Central Hospital Affiliated to Shenyang Medical College, Shenyang, China; 3 Department of Respiratory Medicine, Shengjing Hospital of China Medical University, Shenyang, China

**Keywords:** chronic obstructive pulmonary disease, exosome, cigarette, microRNA, macrophage

## Abstract

Aims: Chronic obstructive pulmonary disease (COPD) is marked by irreversible airflow limitations stemming from small airway constriction and lung emphysema. The advancement of COPD is greatly influenced by the M1 polarization of macrophages. The mechanisms governing macrophage polarization in inflammation conditions in COPD are not yet fully understood.

Methods: To investigate the interplay between exosomes triggered by cigarette smoke and the polarization of macrophages, we utilized a combination of flow cytometry, quantitative real-time reverse transcription PCR, and western blot analysis.

Results: Our research reveals that cigarette smoke (CS) exposure induces the secretion of exosomes from human bronchial epithelial cells, with exosomal miR-221-3p identified as a key player in modulating the polarization of M1 macrophages. The evidence indicates that cigarette smoke promotes exosome secretion in these cells, with exosomal miR-221-3p targeting SOCS3 and regulating the STAT3 signaling pathway to facilitate M1 macrophage polarization.

Conclusions: This research delves into the molecular pathways through which miR-221-3p facilitates the polarization of M1 macrophages, presenting a groundbreaking approach for potential targeted therapy in COPD.

## INTRODUCTION

Chronic obstructive pulmonary disease (COPD), which is mainly caused by cigarette smoking, is characterized by persistent inflammation and impaired tissue repair. This leads to irreversible airflow limitation due to the narrowing of small airways and the onset of lung emphysema [1]. Cigarette smoking plays a crucial role as an environmental factor in the development of COPD [2]. Exposure to cigarette smoke triggers metabolic changes in epithelial cells and the release of bioactive substances into the surrounding microenvironment, resulting in tissue remodeling.

Macrophages play a vital role as a cellular component in the immune microenvironment [3, 4]. They have the ability to differentiate into two different phenotypes, namely classically activated (M1) and alternatively activated (M2), in response to microenvironmental signals. M1 macrophages are recognized for their role in promoting inflammation, while M2 macrophages are linked to inhibiting tumor immune surveillance, promoting tumor invasion, and facilitating tumor growth [5–7]. The interaction between epithelial cells and macrophages remains incompletely understood; elucidating this communication is crucial for enhancing our comprehension of COPD pathogenesis and identifying promising therapeutic targets.

Exosomes are small extracellular vesicles with a diameter ranging from 30 to 100 nm. When examined under cryo-electron microscopy, they exhibit a spherical bilayer structure with a density ranging from 1.13 to 1.19 g/mL [8]. These vesicles contain a variety of biologically active molecules, such as DNA, mRNA, and various non-coding RNAs, including circular RNAs (circRNAs), microRNAs (miRNAs), and long non-coding RNAs (lncRNAs) [9]. The development of COPD is intricately connected to the tissue type and microenvironment of the primary lesion, where genetic variations among diverse tissue cells play a role in the functional diversity of exosomes. Studies have validated that human bronchial epithelial (HBE) cells can release exosomes enriched with miRNA into the tumor microenvironment under hypoxic conditions, resulting in significant regulatory effects [10, 11]. The investigation into the involvement of miRNAs, specifically miR-221-3p, in disease regulation has gained significant attention [12, 13]. Further research is needed to explore whether exosomes carrying miR-221-3p originating from epithelial cells modulate macrophage polarization in an inflammatory microenvironment. Studying the process of M1 macrophage polarization in COPD has become a key focus in the field of immunotherapy [14–17]. Our research specifically examines the impact of cigarette smoke (CS) on M1 macrophage polarization, aiming to provide new perspectives for the development of precise microenvironmental treatments for COPD.

## RESULTS

### Bioinformatics analysis of COPD-related functions

**Figure 1:**
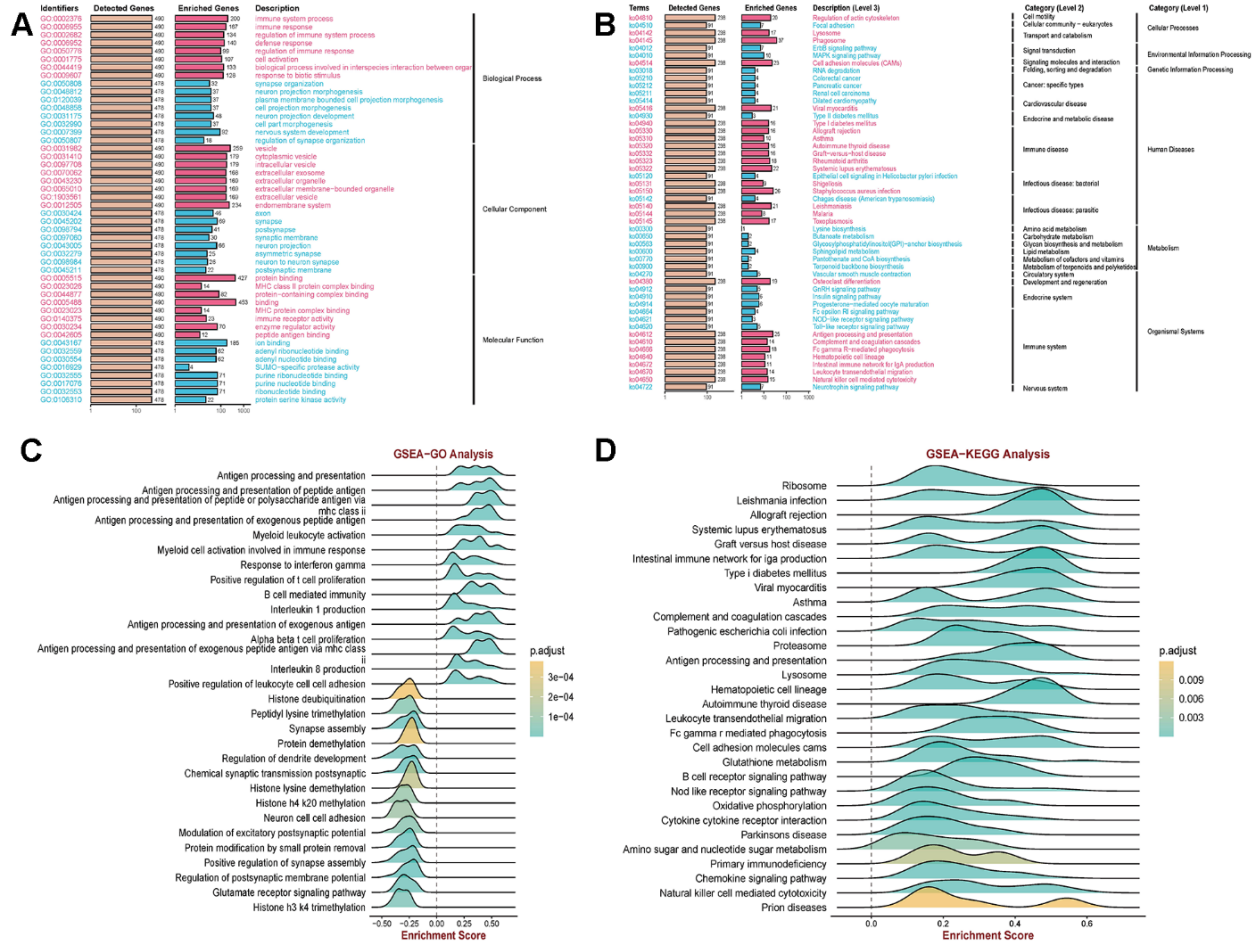
**Clarification of relevant functions in COPD using GEO data.** (**A**, **B**) Kyoto Encyclopedia of Genes and Genomes (KEGG) pathway and Gene Ontology (GO) enrichment analysis revealed that immune pathways (immune system processes; immune response) and vesicle pathways (extracellular exosomes; endosomal system) are associated with COPD. (**C**, **D**) Gene Set Enrichment Analysis (GSEA) demonstrated a significant correlation between COPD and antigen presentation as well as immune response pathways.

This study employed batch effect processing on COPD-related GEO datasets (GSE208662 (32 samples), GSE222965 (24 samples), GSE239897 (82 samples)) obtained from the Gene Expression Omnibus Database (GEO) to validate COPD-associated functions and pathways. The Kyoto Encyclopedia of Genes and Genomes (KEGG) pathway analysis and Gene Ontology (GO) enrichment analysis revealed the significant roles of immune pathways (immune system processes; immune response) and vesicle pathways (extracellular vesicles; endosomal system) in COPD (Figure 1A, 1B). Additionally, Gene Set Enrichment Analysis (GSEA) demonstrated a notable positive correlation between COPD and antigen presentation, as well as immune response pathways (Figure 1C, 1D). These findings underscore the crucial involvement of the immune microenvironment and extracellular vesicles in the development and progression of COPD.

### Cigarette smoke alter the immune microenvironment in COPD

**Figure 2:**
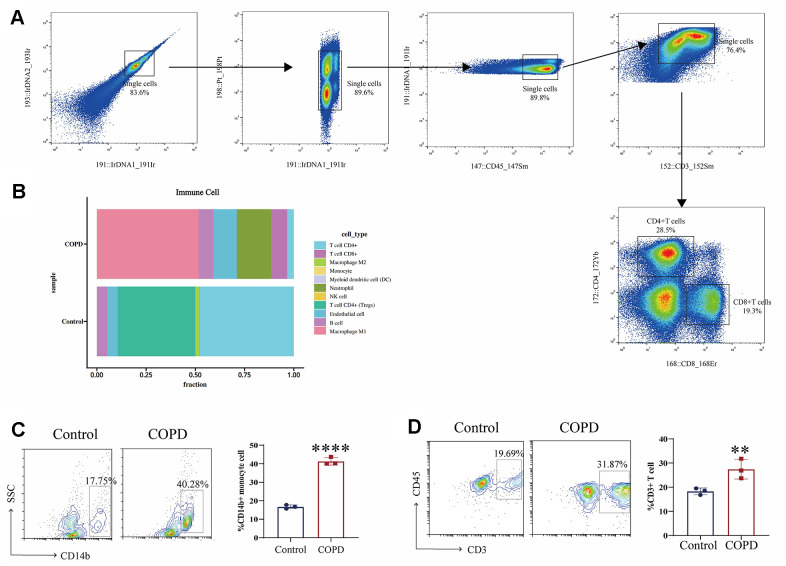
**Immune microenvironment in COPD induced by cigarette smoke.** (**A**, **B**) Mass cytometry was utilized to examine alterations in immune cell composition in the lung tissue of the COPD C57BL/6 mouse model (n = 5 replicates). (**C**, **D**) Moreover, flow cytometry analysis revealed a significant rise in immune cells in the lung tissue of COPD mice. Data are shown as the mean ± SEM (n = 3-5 replicates). *P < 0.05; **P < 0.01; *** *P* < 0.001; ****P < 0.0001.

We investigated the effect of cigarette smoke on the immune microenvironment in chronic obstructive pulmonary disease (COPD) by establishing a COPD mouse model through exposure of C57BL/6 mice to cigarette smoke. Subsequently, mass cytometry was utilized to examine alterations in immune cell composition in the lung tissue of the COPD C57BL/6 mouse model. The results indicated that cigarette smoke exposure elevated the proportions of CD8+ T cells, macrophages, dendritic cells (DCs), and natural killer (NK) cells in the lung tissue of COPD mice, with a notable increase in macrophages (Figure 2A, 2B). Moreover, flow cytometry analysis revealed a significant rise in monocytes in the lung tissue of COPD mice (Figure 2C). Finally, cigarette smoke exposure was associated with an infiltration of CD3^+^T cells in the lung tissue of COPD mice (Figure 2D). These findings underscore the significant changes in monocytes within the lung tissue of COPD mice induced by exposure to cigarette smoke.

### Cigarette smoke enhances exosome secretion from epithelial cells and regulates M1 macrophage polarization

**Figure 3:**
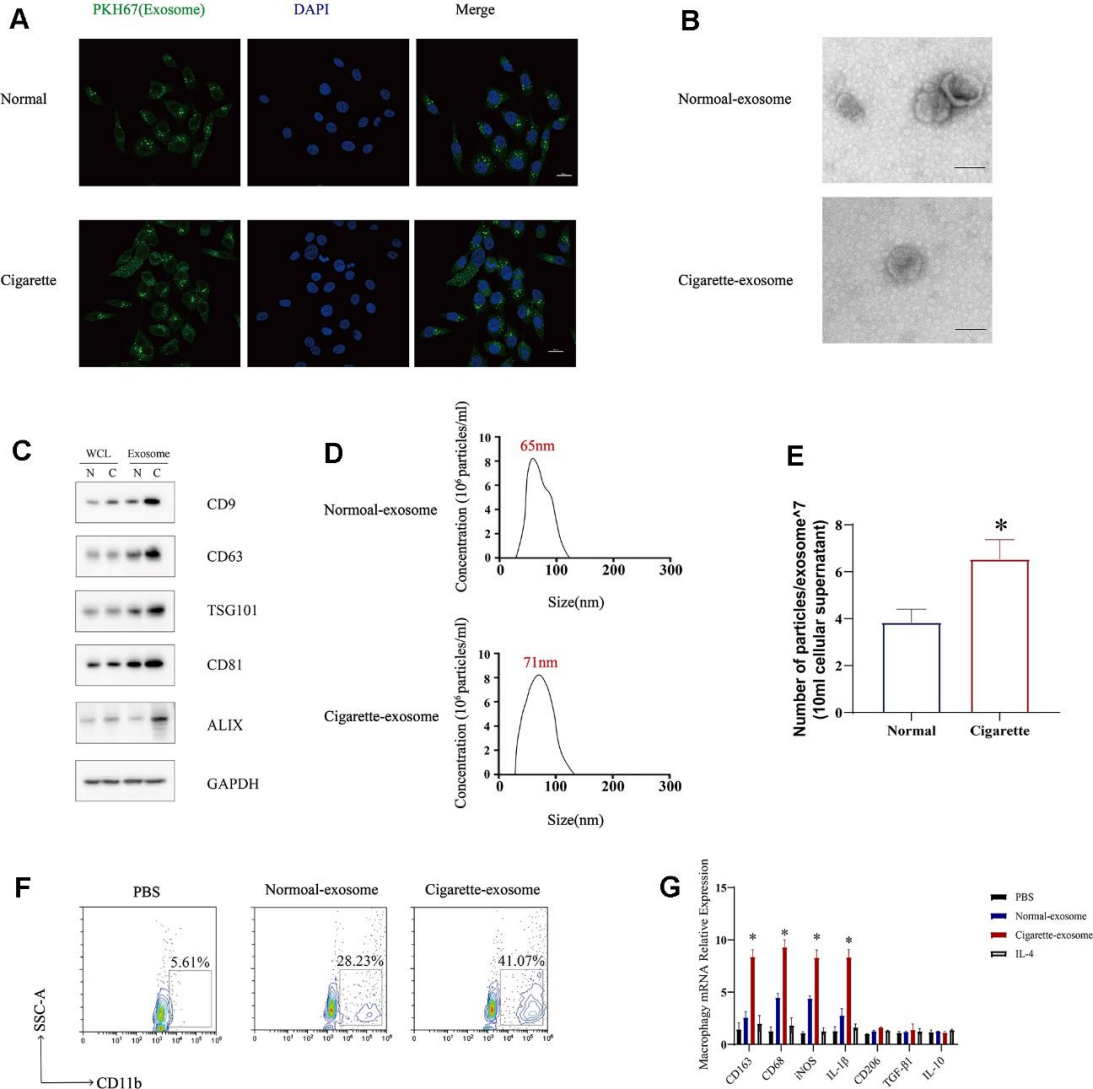
**Exosomes secreted by cigarette smoke (CS)-exposed epithelial cells mediate M1 macrophage polarization.** (**A**) Image showing the internalization of exosomes from CS-epithelial cells immunofluorescently labeled for PKH67 (green). (**B**) Electron micrographs of CS-epithelial cells-produced exosomes purified from conditioned medium from cells grown under Normal (N) and Cigarette (C) conditions. (**C**) Levels of exosomal proteins CD9, CD63, TSG101, CD81, and ALIX in whole cells (WCL) and exosomes extracts determined using western blotting. GAPDH was detected as a loading control. The level of exosomes was evaluated in 1×10^6 cells of cells. (**D**, **E**) The characterization of the purified exosomes was performed using the NanoSight nanoparticle tracking system. (**F**) THP-1 cellular levels of CD11b, a marker of macrophages, detected using flow cytometry. (**G**) Macrophages were treated with N-exosomes, C-exosomes (100 μg/mL) or control (PBS and IL-4). Two days later, the mRNA levels of markers of M2 macrophages (CD206, IL-10 and TGF-β) and markers of M1 macrophages (CD163, CD68, IL-1β and iNOS) were detected using qRT-PCR. Data are shown as the mean ± SEM (n = 3–5 replicates). *P < 0.05; **P < 0.01; *** *P* < 0.001; ****P < 0.0001.

Cigarette smoke has been shown to have an impact on exosomes, which serve as vehicles for biological information [18, 19]. In order to assess the influence of cigarette smoke on the release of exosomes from epithelial cells, we examined the exosomal in both cigarette smoke-exposed and control conditions. The results showed an increase in exosome levels in the presence of cigarette smoke compared to the control group (Figure 3A). Next, the quantification of exosomes within cells was conducted in 1×10^6 cells, with the identification of exosomal markers accomplished in 50 mL of supernatant extracted from an equivalent cell count. Exosomes originating from epithelial cells exposed to cigarette smoke-conditioned medium were examined using electron microscopy, displaying the typical double-layer membrane structure characteristic of exosomes (Figure 3B). Furthermore, the levels of exosome markers, such as CD9, CD63, TSG101, CD81, and ALIX, were evaluated, indicating an increase in exosome secretion triggered by cigarette smoke exposure (Figure 3C). Exosomes were isolated through sucrose gradient density centrifugation, followed by an analysis using nanoparticle tracking analysis (NTA) (Figure 3D, 3E). Our results demonstrated that exposure to cigarette smoke enhances the release of exosomes from lung epithelial cells. Given the essential role of macrophages as primary immune cells in the stromal environment of affected tissues, we investigated the impact of exosomes induced by cigarette smoke on macrophage polarization. The study revealed a distinct polarization of monocytes into M1 macrophages upon exposure to exosomes derived from cigarette smoke, in contrast to those treated with control exosomes (Figure 3F, 3G). As a result, cigarette smoke enhances the release of exosomes from epithelial cells and promotes a shift towards M1 macrophage polarization.

### Transfer of miR-221-3p from exosomes induced by cigarette smoke to macrophages

**Figure 4:**
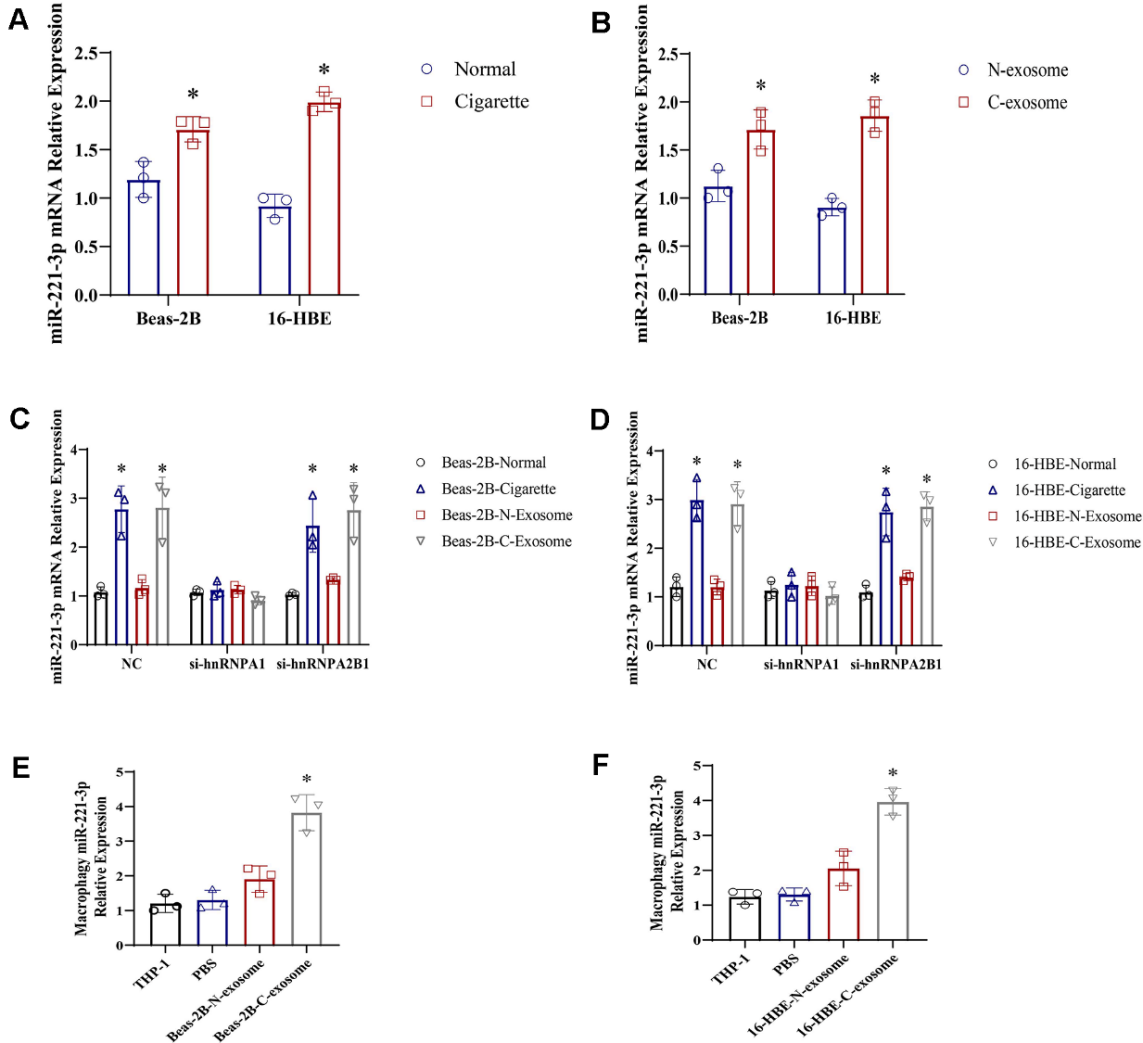
**Exosomes derived from cigarette smoke (CS)-exposed epithelial cells contain high levels of miR-221-3p, which are transferred to macrophages.** (**A**, **B**) qRT-PCR determination of miR-221-3p levels in exosomes from epithelial cells under cigarette conditions. (**C**, **D**) qRT-PCR determination of miR-221-3p levels in exosomes from epithelial cells grown under cigarette conditions following transfection with hnRNPA1 siRNAs. (**E**, **F**) qRT-PCR determination of miR-221-3p levels in macrophages grown in epithelial cells under cigarette conditions subjected to ultracentrifugation to deplete exosomes (exo-free supernatant). Data are shown as the mean ± SEM (n = 3–5 replicates). *P < 0.05; **P < 0.01; *** *P* < 0.001; ****P < 0.0001.

Exosomes are recognized for their packaging of mRNA, miRNA, lncRNA, lipids, and proteins, with a wealth of evidence supporting the presence of noncoding RNAs [20, 21]. Specifically, miR-221-3p has been detected within these vesicles [13]. To confirm the increased presence of miR-221-3p in exosomes derived from epithelial cells exposed to cigarette smoke, a quantitative real-time polymerase chain reaction (qRT-PCR) analysis was performed. The findings revealed higher levels of miR-221-3p in both cigarette smoke-exposed epithelial cells and the exosomes derived from them, in comparison to the control group (Figure 4A, 4B). To assess whether the incorporation of miR-221-3p into exosomes is facilitated by hnRNPA1 in the presence of smoke exposure, small interfering RNAs (siRNAs) targeting hnRNPA1 were employed. Upon silencing hnRNPA1, a significant decrease in the levels of exosomal miR-221-3p was noted under smoke conditions (Figure 4C, 4D), suggesting the participation of hnRNPA1 in this mechanism. Further exploration examined the ability of exosomes induced by smoke to deliver miR-221-3p to macrophages. Treatment of macrophages with these exosomes resulted in an increase in the accumulation of miR- 221-3p (Figure 4E, 4F), validating the transfer of miR-221-3p from cigarette smoke-exposed epithelial cells to macrophages via exosomes.

### Cigarette smoke-induced hnRNPA1 facilitates the sorting of miR-221-3p into exosomes

**Figure 5:**
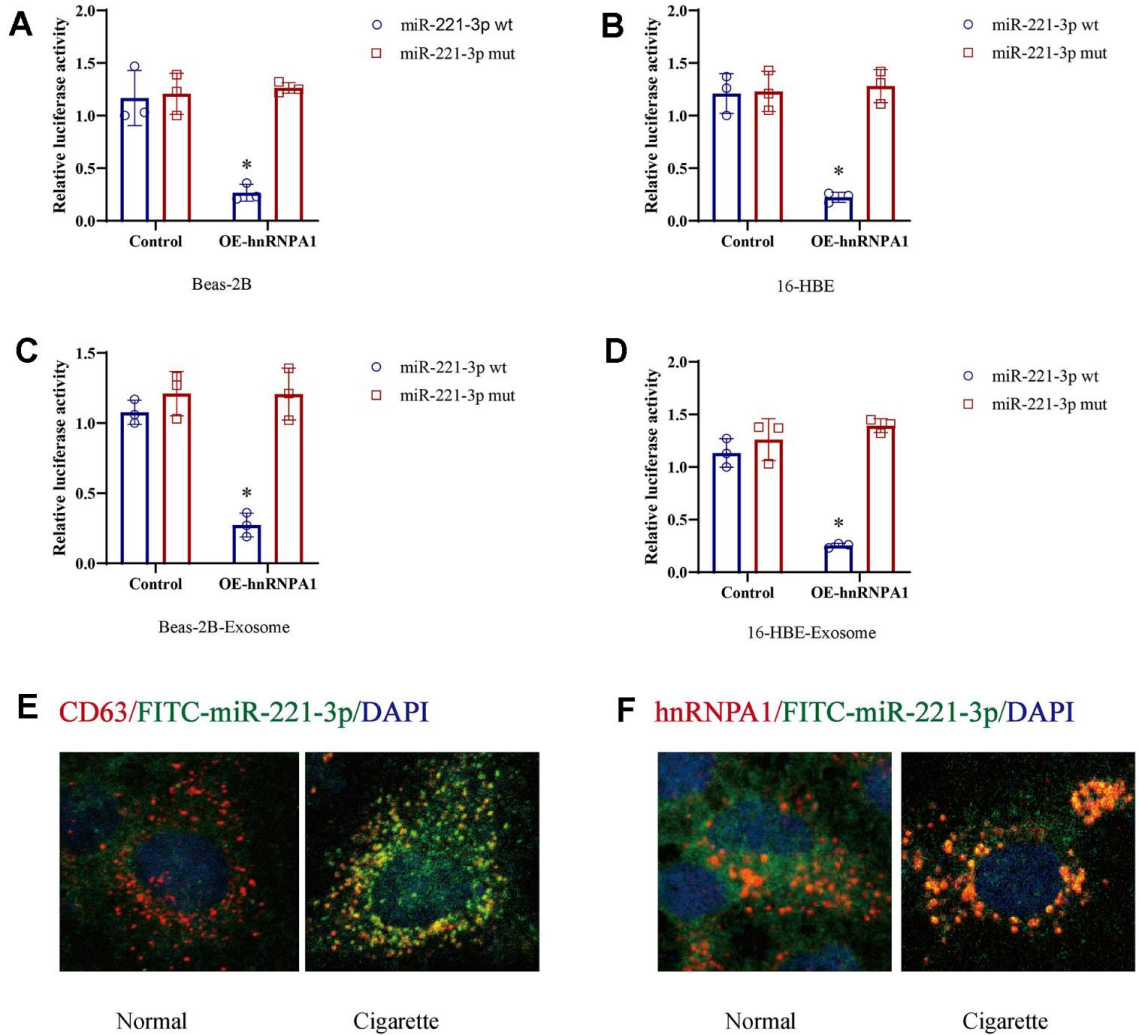
**Cigarette smoke (CS) induces exosomal miR-221-3p through binding with hnRNPA1.** (**A**–**D**) CS-epithelial cells were co-transfected with wild-type or mutant miR-221-3p and hnRNPA1 and then subjected to luciferase reporter assays. The ratio of Renilla luciferase signals to firefly luciferase signals was used to normalize the luciferase activity at 48 h post-transfection. (**E**, **F**) The co-location of CD63, hnRNPA1 and miR-221-3p using immunofluorescence.

Previous studies have suggested that the RNA-binding protein hnRNPA1 is involved in the packaging of miRNAs into exosomes. This study aimed to determine if miR-221-3p is a specific target of hnRNPA1 by analyzing the presence of hnRNPA1 consensus binding sites within the miR-221-3p sequence. Using luciferase reporter assays, we found that co-transfection of wild-type miR-221-3p with hnRNPA1 in cells exposed to cigarette smoke or their secreted exosomes resulted in a substantial increase in luciferase activity, an effect not replicated with a mutated variant of miR-221-3p (Figure 5A–5D). In the presence of cigarette smoke, a progressive increase in the co-localization of exosomal markers CD63, hnRNPA1, and miR-221-3p was observed (Figure 5E, 5F). These results indicate that cigarette smoke elevates exosomal levels of miR-221-3p through an hnRNPA1-dependent mechanism.

### Exosomes induced by cigarette smoke enhance M1 macrophage differentiation via miR-221-3p

**Figure 6:**
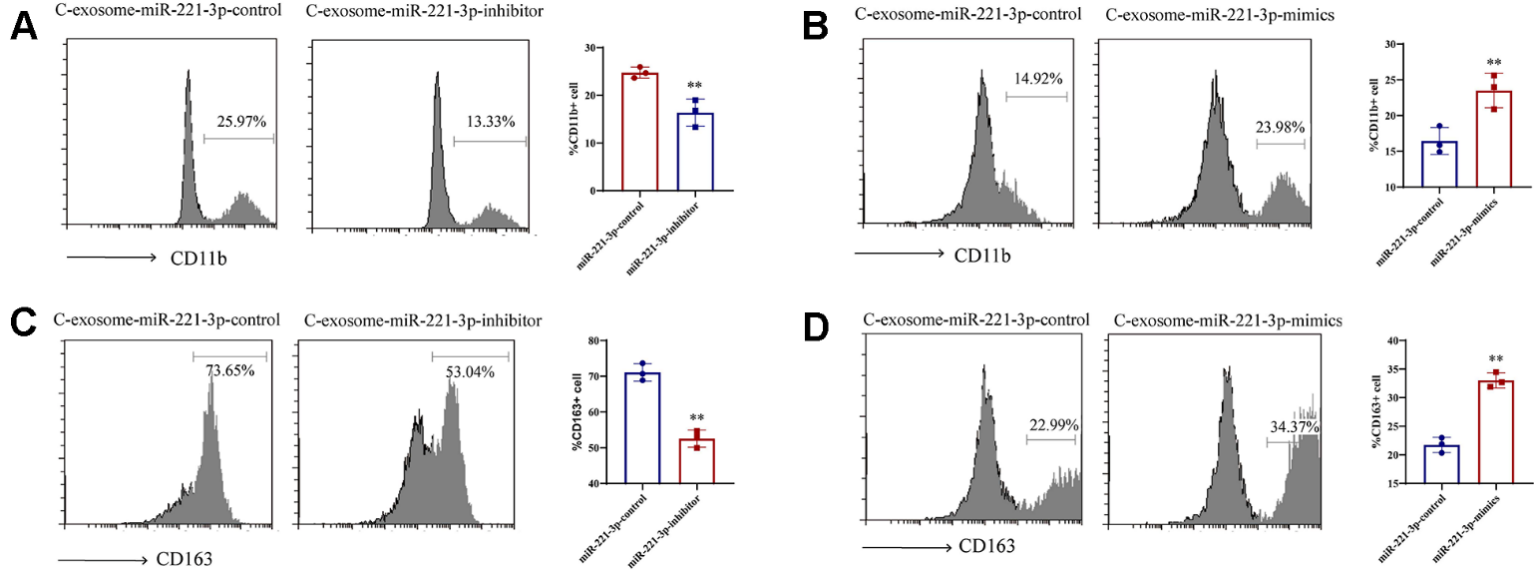
**Induction of M1 macrophage differentiation by exosomal miR-221-3p from cigarette smoke (CS)-exposed cells.** (**A**, **B**) Numbers of CD11b^+^ monocytes in response to cigarette smoke-induced exosomes miR-221-3p treated with epithelial cells, as assessed using flow cytometry. (**C**, **D**) Numbers of CD163^+^ macrophages in response to cigarette smoke-induced exosomes miR-221-3p, as assessed using flow cytometry. Data are shown as the mean ± SEM (n = 3–5 replicates). *P < 0.05; **P < 0.01; *** *P* < 0.001; ****P < 0.0001.

Moreover, to elucidate the interaction between cigarette smoke-induced exosomes and monocytes, microscopic analysis was utilized. Treatment of THP-1 cells with these exosomes led to an upsurge in the accumulation of CD14^+^ monocytes, in contrast to those treated with exosomes from which miR-221-3p had been silenced (Figure 6A, 6B). Additionally, miR-221-3p packaged within exosomes derived from cigarette smoke increased the expression of CD163^+^macrophages (Figure 6C, 6D), as demonstrated by the enhanced formation of M1 macrophages and expansion of their network.

### Exosomal miR-221-3p derived from cigarette smoke exerts regulatory control over M1 macrophage polarization through the activation of the STAT3 signaling pathway

**Figure 7:**
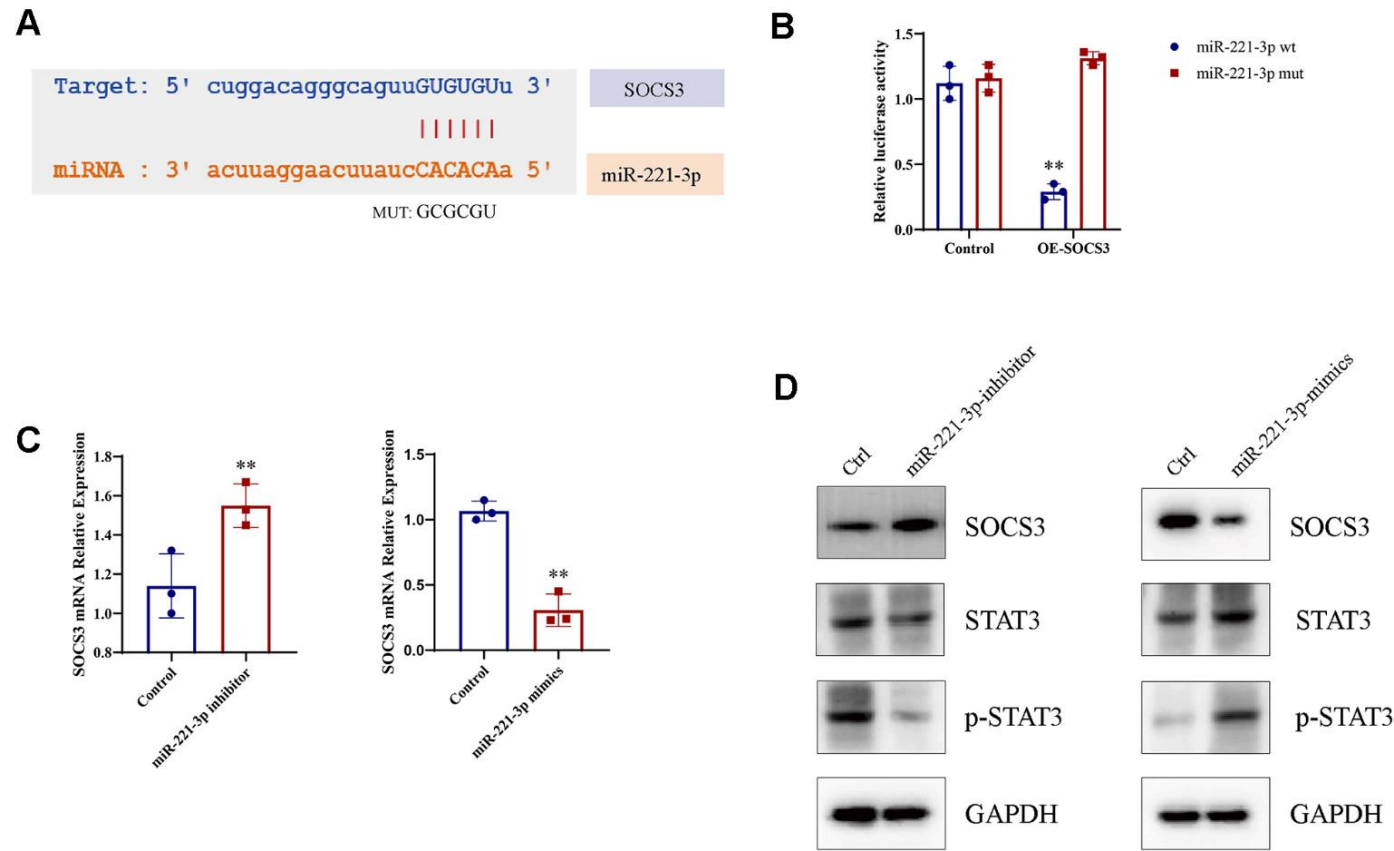
**Macrophage M1 polarization is enhanced by miR-221-3p from exosomes induced by cigarette smoke (CS), leading to increased p-STAT3 expression.** (**A**) Binding sites for miR-221-3p in SOCS3 using microRNA.org-Targets3. (**B**) Luciferase reporter assays in epithelial cells co-transfected with wild-type or mutant miR-221-3p and SOCS3. (**C**) qRT-PCR determination of SOCS3 expression in epithelial cells transfected with miR-221-3p inhibitors or miR-221-3p mimics. (**D**) STAT3 levels in macrophages transfected with miR-221-3p inhibitors or miR-221-3p mimics determined using western blotting. Data are shown as the mean ± SEM (n = 3–5 replicates). *P < 0.05; **P < 0.01; *** *P* < 0.001; ****P < 0.0001.

In order to clarify the molecular mechanism by which exosomal miR-221-3p from cigarette smoke-exposed cells promotes M1 macrophage polarization, we searched for potential miR-221-3p binding sites on *SOCS3* mRNA using the microRNA.org-Targets database (Figure 7A). Luciferase assays validated that co-transfection with wild-type miR-221-3p resulted in decreased luciferase activity in constructs harboring *SOCS3* sequences (Figure 7B). Moreover, overexpression of miR-221-3p suppresses *SOCS3* mRNA expression levels in macrophages (Figure 7C), concomitant with increased phosphorylation of STAT3 (p-STAT3) (Figure 7D). Overall, these *in vitro* findings collectively illustrate that miR-221-3p derived from exosomes released by cells exposed to cigarette smoke regulated SOCS/STAT3 signaling pathways, consequently fostering M1 polarization in macrophages.

## DISCUSSION

Chronic obstructive pulmonary disease (COPD) is a significant worldwide health challenge, as its prevalence and related morbidity and mortality are increasing. Persistent airway inflammation is a key characteristic of COPD, contributing significantly to its development and advancement. However, anti-inflammatory therapies are generally not the primary approach in managing this condition [22]. Recent findings posit that cigarette smoke modulates the release of exosomes by epithelial cells, which are integral to inflammatory processes, suggesting that smoke may drive exosome secretion in these cells [23]. This study identified exosomes derived from epithelial cells following exposure to cigarette smoke and illuminated their contribution and underlying mechanism in promoting COPD progression.

New evidence suggests that exosomes can create an immunosuppressive environment. Macrophages are essential in therapy, particularly M1 macrophages for controlling inflammation [24, 25]. This study validated that exosomes triggered by cigarette smoke support the induction of M1 macrophages. The development of COPD is influenced by the SOCS3/STAT3 signaling pathway, which regulates inflammatory reactions [26–29]. Activation of this pathway by miR-221-3p, carried within exosomes induced by cigarette smoke, was observed to enhance the polarization and differentiation of M1 macrophages. Our findings suggest that exosomes derived from epithelial cells enhance macrophage STAT3 signaling. In this process, miR-221-3p inhibits SOCS3 expression by binding to its 3′UTR. Furthermore, the overexpression of miR-221-3p was linked to elevated STAT3 expression and phosphorylated STAT3 (p-STAT3) levels in macrophages. In conclusion, our investigation delves into the role of cigarette smoke in amplifying exosome release from epithelial cells and delineates how these exosomes steer M1 macrophage polarization.

Expanding on our prior research, we propose the following scientific hypothesis: cigarette smoke boosts the release of exosomes from epithelial cells. These exosomes transport miR-221-3p to macrophages, influencing the SOCS3/STAT3 signaling pathways and ultimately driving the polarization of M1 macrophages.

## MATERIALS AND METHODS

### Bioinformatics analysis

The Gene Expression Omnibus Database (GEO database) was utilized to download single-cell data related to Chronic Obstructive Pulmonary Disease (COPD), including datasets GSE208662, GSE222965, and GSE239897. Statistical methods, such as edgeR and DESeq2 software packages, were applied to identify differentially expressed genes. To address the issue of false positive errors stemming from multiple hypothesis testing, the Benjamini-Hochberg method was employed to correct the false discovery rate (FDR).

### Functional enrichment and annotation of GO/KEGG pathways

Differentially expressed genes were analyzed for gene function enrichment and annotation using the DAVID database (https://david.ncifcrf.gov/home.jsp). This analysis aimed to investigate the enrichment of differentially expressed genes in various pathways and cellular functions by leveraging the Gene Ontology (GO) and Kyoto Encyclopedia of Genes and Genomes (KEGG) databases. Particularly, genes relevant to the COPD microenvironment and other significant aspects were further explored.

### GSEA analysis

For the Gene Set Enrichment Analysis (GSEA), software such as GSEA or R packages like clusterProfiler were utilized. This analysis involved comparing a ranked gene list with predefined gene sets. The GSEA algorithm calculated an Enrichment Score (ES) to evaluate the distribution of gene sets within the ranked list, where a higher ES indicated a stronger association with a specific phenotype. To determine the statistical significance of the observed ES, GSEA performed multiple random permutation tests to derive nominal p-values and false discovery rates (FDR) that indicated the significance of gene set enrichment.

### Preparation of cigarette smoke extract (CS)

In short, the aerosol (containing 10 mg of tar, 0.8 mg of nicotine in cigarette smoke, and 11 mg of carbon monoxide in cigarette smoke) generated from a 3R4F Research Cigarette (University of Kentucky, USA) was passed through a flask filled with 10 mL of warmed (37° C) minimum essential medium (MEM) using a consistent speed vacuum pump, with each cigarette undergoing a 5-minute smoking period. The CS solution was pH-adjusted to 7.4 and subsequently sterilized via filtration using a 0.22-μm pore filter (Schleicher and Schuell GmbH, Dassel, Germany). To ensure quality control, the solution underwent standardization by measuring absorbance at 320 nm (A320) and 540 nm (A540). The CS quality met the criteria if the difference in optical density (ΔOD = A320 - A540) fell within the range of 0.9 to 1.2. The resulting CS solution, considered to be a 100% CSE, was diluted with medium for use in experiments within one hour.

### Cell lines

Human bronchial epithelial cells (HBE-16 and Beas-2B) were sourced from the Shanghai Institute of Cell Biology, Chinese Academy of Sciences. The cells were cultured in minimum essential medium (MEM) supplemented with 10% fetal bovine serum (FBS) from Thermo Fisher Scientific, Waltham, MA, USA, and 100 mg/mL streptomycin along with 100 U/mL penicillin, also from Thermo Fisher Scientific, Waltham, MA, USA. The cells were incubated in a 5% CO_2_ atmosphere at 37° C. The human monocyte cell line THP-1 was acquired from the Chinese Academy of Sciences in Shanghai, China. The cells were cultured in Roswell Park Memorial Institute (RPMI) 1640 medium obtained from Gibco in Grand Island, NY, USA, supplemented with 10% fetal bovine serum (FBS) also from Gibco. Culturing of the cells was conducted in a humidified environment with 5% CO2 at 37° C. To induce macrophage differentiation, THP-1 cells (3×10^5) were seeded onto 0.4 μm pore inserts and treated for 24 hours with 200 nM phorbol-12-myristate-13-acetate (PMA) sourced from Sigma-Aldrich in St. Louis, MO, USA. The non-contact co-culture Transwell system from Corning Inc., located in Corning, NY, USA, was utilized for the co-cultivation of cells from COPD and macrophages. THP-1 inserts were placed in a 6-well plate previously seeded with 1 × 10^5 human bronchial epithelial cells per well, and the co-culture was maintained for 48 hours. The cell cultures were incubated in a 5% CO_2_ environment at 37° C.

### Western blotting

Protein samples were extracted by cell lysis using Radioimmunoprecipitation assay (RIPA) lysis buffer (Cat. No. P0013, Beyotime, Jiangsu, China). After separation by 12% sodium dodecyl sulfate-polyacrylamide gel electrophoresis (SDS-PAGE), the proteins were electrophoretically transferred to a polyvinylidene fluoride (PVDF) membrane. The PVDF membrane was incubated in 5% BSA at room temperature for 2 h, and then at 4° C overnight with primary antibodies recognizing the following proteins: CD9 (1:1000; CST, USA, Rabbit, #13403), TSG101 (1:1000; Proteintech, China, Rabbit, #28283–1-AP), CD63 (1:1000; GeneTex, USA, Rabbit, #GTX28219), ALIX (1:1000; CST, Rabbit, #92880), CD81 (1:1000; ABclonal, USA, Rabbit, #A5270), suppressor of cytokine signaling 3 (SOSC3) (1:1000, CST, Rabbit, #52113), STAT3 (1:1000; CST, Rabbit, #9139), phosphorylated (p)-STAT3 (1:1000; CST, Rabbit, #9154), GAPDH (1:1000; CST, Rabbit, #5174), and then incubated with horseradish peroxidase (HRP)-conjugated antibodies for 2h at room temperature for visualization using enhanced chemiluminescence (ECL).

### Immunofluorescence

Epithelial cells altered by exposure to cigarette smoke (CS) were cultivated on glass coverslips and subsequently treated with 4% paraformaldehyde at ambient temperature for a duration of 15 minutes. The fixed cells were rinsed with phosphate-buffered saline (PBS) containing 0.2% Triton X-100, followed by blocking with 1% bovine serum albumin (BSA) and overnight incubation at 4° C with primary antibodies. The primary antibodies recognized: CD63 (1:150, Abcam, UK, Mouse, #Ab1318), hnRNPA1 (1:150, Abcam, Mouse, #Ab5832). The cells were washed three times using PBS, and then incubated with secondary antibodies at a 1:1000 dilution, including Alexa Fluor 594 goat anti-rabbit IgG (1:10000; Life Technologies, USA, A11012), Alexa Fluor 488 goat-anti mouse IgG (1:10000; Life Technologies, A11029). The cells were washed three times using PBS and then the nuclei were stained with 4',6-diamidino-2-phenylindole (DAPI), placed on slides, and observed under a Nikon A1R confocal microscope with a 20× objective (Nikon, Tokyo, Japan).

### Purification of the extracellular vesicles

Exosomes secreted by cells were obtained from cell supernatant by incubating the cells in a medium supplemented with 10% exosome-depleted FBS. Standard differential centrifugation was utilized to purify extracellular vesicles from the supernatants of cell cultures maintained for 48 to 72 hours. To eliminate cell debris and dead cells, cell culture supernatants underwent centrifugation at 2000 g for 20 minutes. Following centrifugation at 17,000 g for 40 minutes, the microvesicles were reconstituted. The resultant supernatant underwent ultracentrifugation at 100,000 g for 2 hours at 4° C. The residual material was resuspended in PBS before undergoing a final ultracentrifugation cycle at 100,000 g for another 2 hours.

### Isolation of exosomes by sucrose gradient centrifugation

The exosomes that were procured by means of differential ultracentrifugation underwent a high-speed centrifugation process at 100,000 g for a span of 2.5 hours. Subsequent to collection, these exosomes were submerged individually in sucrose solutions of varying densities, specifically 10-16%, 22-28%, 34-40%, 46-52%, 58-64%, and 70-82%. Each fraction therein was diluted in the ratio 1:100, and re-suspended at 100,000 g, followed by another round of centrifugation for 2.5 hours in PBS, prior to undertaking western blot. Qualitative analysis of these samples was realized using western blot, and corroborated further with a western blot technique that employed exosome protein antibodies.

### Characterization of the purified exosomes

To ascertain the size distribution of exosomes, an initial step of diluting the exosomes tenfold using a 0.22 μm filter membrane is essential to gain an optimal observation count. Post dilution, 30 μl of diluted exosomes are applied onto a carbon-coated copper mesh that is positioned atop a sealed membrane, allowed to settle for between 2 to 5 minutes. Consequently, the supporting membrane is left to dry after being stained with a uranium acetate dye solution for a duration of 90 seconds. The stained support membrane is then placed on filter paper to dry for a subsequent 3 hours in preparation for observation. Ultimately, size measurement and separation of exosomes are performed utilizing the NanoSight NS300 instrument (Malvern Instruments Ltd., Worcestershire, UK), with the database being analyzed through NTA software (NTA version 2.3).

### Isolation and culture of human CD3^+^ T cells

Flow cytometry was used to sort CD3^+^ T cells from peripheral blood mononuclear cells (PBMCs) from healthy individuals using FACSAria Fusion (BD Biosciences, San Jose, CA, USA). Anti-CD3 monoclonal antibodies (mAbs) (clone: OKT3) (eBioscience, San Diego, CA, USA; 16-0037-81), anti-CD28 mAbs (clone: CD28.2) (eBioscience, 14-0289-82), and transforming growth factor beta 1 (TGF-β1 (MCE, Monmouth Junction, NJ, USA; A279-S390) were used to stimulate sorted CD3^+^ T cells, which were grown in RPMI-1640 medium with 10% FBS and 150 IU/mL of recombinant human interleukin-2 (IL-2) (Novoprotein, Shanghai, China).

### Flow cytometric and mass cytometry

A FACS Calibur (BD Biosciences, USA) and mass cytometry were employed for COPD C57BL/6 mice. After dissociation into single-cell suspensions, lung cells were rinsed prior to incubation within the staining solution that contained 2 mE EDTA and 1% BSA using appropriate fluorescent monoclonal antibodies or corresponding isotype controls for a 30-min period under 4° C. Antibodies were directly conjugated with FITC, PE, APC, or if unavailable. The monoclonal antibodies were tagged with fluorescein isothiocyanate (FITC) and results were calculated using Cell Quest software (BD Biosciences). The following antibodies (clones) were purchased from BD Biosciences: CD11b (741138), CD11c (741139), CD45 (741616), MHC-II (567852), CD3 (557596), NK1.1 (568064), Granzyme B (372208), Perforin (563576), CD14 (551403), CD206 (551135), and CD163 (752069).

### Quantitative real-time reverse transcription PCR

Total RNA was extracted using the TRIzol reagent (Thermo Scientific, Waltham, MA, USA). The RNA was reverse-transcribed to cDNA using the SuperPrep II Cell Lysis & RT kit for qPCR (Toyobo, Osaka, Japan). The qPCR reactions were performed using a 7500 Fast DX Real-Time PCR system (Applied Biosystems, Foster City, CA, USA). The threshold cycle (Ct) method was used to determine the expression levels. Three replicate tests were performed for each sample, and glyceraldehyde-3-phosphate dehydrogenase (GAPDH) was detected as an internal reference.

*hsa-miR-221-3p*

*F:GACTAGCTACATTGTCTGCTG*

*R:GTCGTATCCAGTGCAGGGTCCGAGGTATTCGCACT GGATACGACGAAACC*

*CD163 F: GGGATGTCCAACTGCTATCAA R: GACTCATTCCCACGACAAGAA*

*CD68 F: GCTTTGCAATCTCCCTGTTG R: TTGATCCGGGTTCTTACCTG*

*iNOS F: CTGATCTTGTGCTGGAGGTGACC R: TTGAAGGGGCAGGCTGGG*

*IL-1β F: AAGAAGAACGGAAGAATCAG R: CAGATATACAGGGAGTCACC*

*TGF-β1 F: GGCCAGATCCTGTCCAAGC R: GTGGGTTTCCACCATTAGCAC*

*CD206 F: GGACGTGGCTGTGGATAAAT R: ACCCAGAAGACGCATGTAAAG*

*IL-10 F: TCTCCGAGATGCCTTCAGCAGA R: TCAGACAAGGCTTGGCAACCCA*

*SOCS3 F: CGGACCTGGAATGTGTTGGA R: TCAGCATTCCCGAAGTGTCC*

*GAPDH F: TGTTGCCATCAATGACCCCTT R: TCCACGACGTACTCAGCG*

### Establishment of COPD animal model

Forty C57BL/6 mice aged 6–8 weeks were randomly divided into the Control group (non-smoking control group, n = 10) and Model group (smoking group, n = 10). In each experiment, age and sex were matched in each group. Among them, the mice exposed to the air served as the Control group. Mice in the Model group were exposed to CS. Mice were exposed in a barrier facility using a systemic exposure system (SCIREQ “inexposure”). After the last treatment, the mice were euthanized by cervical dislocation after intraperitoneal injection of pentobarbital (150 mg/kg) and whole lung tissues were collected. The experiments on animals were approved by China Medical University and all methods were carried out in accordance with the ARRIVE guidelines and relevant regulations.

### Statistics

SPSS statistical software (version 22.0, IBM Corp., Armonk, NY, USA) and GraphPad Prism software (version 6.0, GraphPad Inc., La Jolla, CA, USA) for Windows were used to carry out all the statistical analyses. All experiments were carried out at least in triplicate and analysis of variance (ANOVA) was used to calculate the statistical significance. The median of all groups with *P* < 0.05 was compared using nonparametric tests for ANOVA.

### Availability of data and materials

The datasets used and/or analyzed during the present study are available from the corresponding author on reasonable request.

### Consent for publication

All authors agree to submit the article for publication.
